# Lymphoepithelial cyst as a herald of HIV seropositivity in a patient with known history of neurocysticercosis and suspected parotid cysticercosis

**DOI:** 10.1259/bjrcr.20150119

**Published:** 2015-01-19

**Authors:** Alan Alexander, Kyle Hunter, Daniel Wasdahl, Michael Markovic

**Affiliations:** ^1^ Department of Radiology, Aultman Hospital, Canton, OH, USA; ^2^ Department of Pathology, Aultman Hospital, Canton, OH, USA

## Abstract

Among those with acquired immune deficiency syndrome, salivary gland pathology and other less common signs of human immunodeficiency virus (HIV) seropositivity are emerging. Generally speaking, lymphoepithelial lesions of the parotid gland are uncommon with a reported incidence of 0.6%, but they are beginning to overtake other oral lesions such as candidiasis as predominant oral manifestations of clinical HIV infection. Here, we describe a patient with a known history of neurocysticercosis with presumed extracranial cysticercosis as demonstrated by the clinical manifestation of bilateral parotid gland swelling and a ring-enhancing, hypodense lesion of the left parotid gland on CT. He was found to have a lymphoepithelial cyst arising in a lobe of the left parotid gland per pathological evaluation after left superficial parotidectomy, and this served as the initial sign of HIV positivity, which was confirmed by serological studies.

## Summary

Among those with untreated acquired immune deficiency syndrome (AIDS), salivary gland pathology and other less common signs of human immunodeficiency virus (HIV) seropositivity are emerging.^1^ Generally speaking, lymphoepithelial lesions of the parotid gland are uncommon with a reported incidence of 0.6%, but they are beginning to overtake other oral lesions such as candidiasis as predominant oral manifestations of untreated clinical HIV infection.^1,2^ Here, we describe a patient with a known history of neurocysticercosis with presumed extracranial cysticercosis, as demonstrated by the clinical manifestation of bilateral parotid gland swelling and a ring-enhancing, hypodense lesion of the left parotid gland on CT. He was found to have a lymphoepithelial cyst arising in a lobe of the left parotid gland per pathological evaluation after-left superficial parotidectomy, and this served as the initial sign of HIV positivity, which was confirmed by serological studies.

## Background

Bhaskar and Bernier^3^ first introduced the term “lymphoepithelial cyst” in 1958 as a replacement for what had formerly been called “branchial cleft cyst,” as they concluded that the parotid lesions they observed had originated from parotid inclusions within lymph nodes rather than embryonal remnants. The origin of these cysts still remains a topic of controversy, and several theories have been posed.^4^ It is hypothesized that the development of lymphoepithelial cysts as benign lymphoepithelial lesions (BLEL) may represent a localized manifestation of persistent generalized lymphadenopathy, which is commonly seen with HIV infection and autoimmune entities such as Sjögren’s syndrome and sarcoidosis. Alternatively, this parotid pathology may be secondary to lymphatic infiltration of salivary parenchyma, which promotes basal cell hyperplasia of the striated ducts and formation of the lymphoepithelial lesion.^1^

BLEL of the parotid salivary gland were first described in 1985.^2^ These lesions may be solitary or multifocal, cystic, solid, or heterogeneous lesions that may occur either unilaterally or, as is more frequently described with concomitant HIV, bilaterally.^4,5^ Parotid swelling is most frequently accompanied by cervical lymphadenopathy in BLEL.^6^ Although benign and painless, these parotid lesions enlarge over time and may become disfiguring if not treated appropriately.^5^BLEL are commonly associated with HIV infection, although they are not necessarily considered precursors to fulminant AIDS.^1^ Lymphoepithelial cysts may also arise secondary to pathogenetic processes independent of HIV infection.^4^Still, the presence of these indolent lesions has frequently been described as the first sign of HIV infection. BLEL have been documented in 5–10% of HIV-positive patients and in up to 20% of patients with AIDS.^1,4^ In addition, the viral load in HIV infection shows linear association with gland enlargement secondary to BLEL.^1^

## Imaging and pathological evaluation

Initial imaging evaluation of BLEL is most commonly conducted with ultrasonography and CT scan. The lesions may range from nearly completely fluid-filled masses to those of mixed composition with solid components. For the sake of simplicity, a dichotomous sonographic classification scheme according to echogenicity, as proposed by Martinoli et al,^6^ may be employed for the evaluation of BLEL of both cyst and mixed types. The majority of cyst-type lesions are round or ovoid in morphology, with scattered isolated internal echoes. Compression of these lesions tends to suggest fine, filamentous septations. Mixed-type lesions tend to have more irregular, lobulated morphologies. They demonstrate moderate echogenicity to hyperechogenic matrix with solid component predominance, resembling a solid tumour. However, with compression, the motion of internal echoes indicate at least partial fluid composition. Vascularity, as assessed with colour Doppler, is variable across these two subtypes; some are relatively avascular, while others demonstrate hypervascularity. Overall, sonographic appearance of BLEL closely resembles that of parotid glands in Sjögren’s syndrome or Warthin’s tumour, although parotid cysts in Sjögren’s syndrome tend to be smaller and more numerous. BLEL may be differentiated from Warthin’s tumour by the presence of cervical lymphadenopathy.^6^

As the spectrum of sonographic findings of BLEL are non-specific, especially with regard to heterogeneous solid cystic lesions, contrast-enhanced CT scan offers more diagnostic power.^6^ Findings typical of BLEL, as seen on contrast-enhanced CT scan, include numerous mixed cystic and solid lesions in either one or bilateral parotid glands. Rarely, a solitary cystic lesion may represent a lymphoepithelial cyst. A rim of peripheral enhancement usually encircles the hypodensities, suggestive of cystic components, while heterogeneous enhancement comprises solid components. In addition, the neck should be surveyed for non-necrotic cervical lymphadenopathy as well as tonsillar hypertrophic changes, as these strengthen the case for BLEL.^7^

MRI findings include intraparotid cystic lesions with low heterogeneous signal intensity on *T*_1_ weighted images, and high signal intensity on *T*_2_ weighted images. In addition, *T*_2_ hyperintense bilateral cervical adenopathy is often present. Similar to CT scan, prominence of the Waldeyer's lymphatic ring with high *T*_2_ signal intensity is also suggestive of BLEL in the setting of the intraparotid findings. Contrast enhancement demonstrates thin enhancing rims of intraparotid cystic lesions with variable enhancement of solid lesions.^8^

Differential diagnosis includes parotid cysticercosis, lymphoma, metastatic disease, Warthin tumour, parotid sarcoidosis and Sjögren’s syndrome. Sjögren’s syndrome and sarcoidosis can appear identical to BLEL on imaging, and attention is paramount to the aforementioned ancillary findings. Warthin tumours lack the ancillary findings of tonsillar hyperplasia. Lymphoma often has additional clinical symptoms or multifocal findings. Metastatic disease is uncommon bilaterally. On CT scan and MRI, parotid cysticercosis appears as a cystic structure with surrounding oedema in the colloidal stage, and a thickened retracted cyst with decreased oedema in the granular stage, both appearing similar to BLEL. The wall of the cyst will enhance with contrast administration, although often there is nodular/marginal enhancement secondary to the scolex.^9^

The diagnosis of BLEL is made by pathological evaluation. Histopathologically, BLEL are characterized by follicular hyperplasia, ectasia of terminal striated ducts and lymphocytic infiltration. Fine needle aspirate cytology of BLEL reveals a characteristic triad of heterogeneous lymphoid aggregates, foamy macrophages of varying number and distribution, and superficial and/or enucleated squamous cells. In addition, the presence of multinucleated giant cells within lymphoepithelial cysts has been described.^5^Among large lymphoepithelial cysts, similar immunohistochemical profiles with regard to expression of S100 have been observed, regardless of HIV seropositivity. BLEL related to HIV infection may be further delineated by positivity for p24 expression.^10^

## Case report

**Figure 1.**
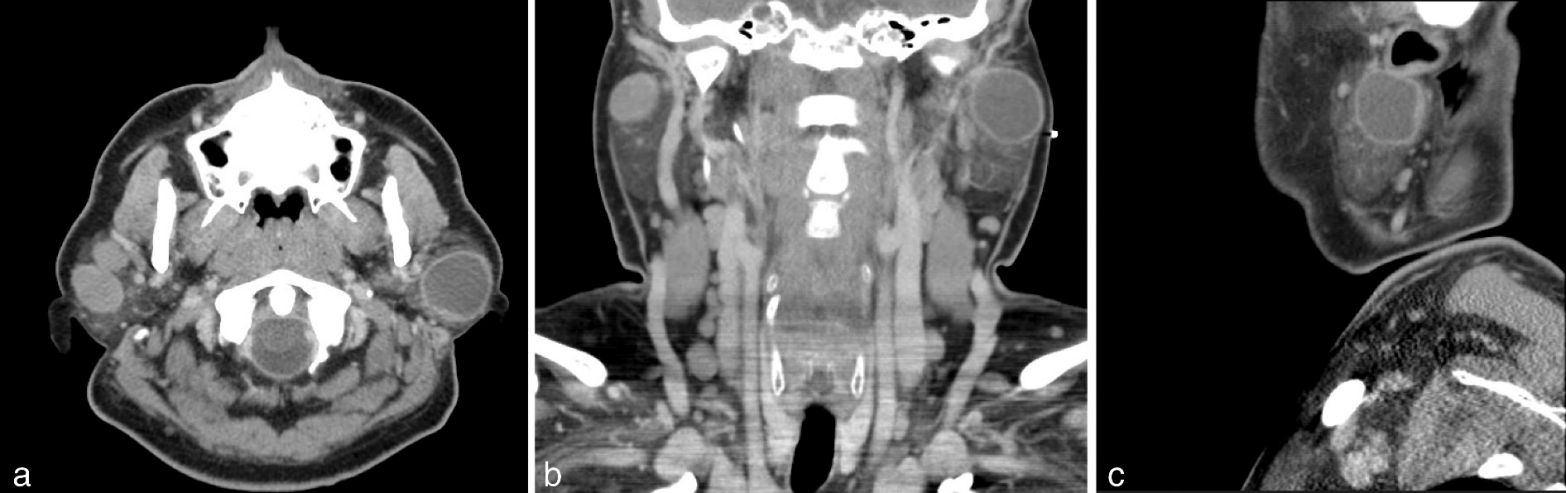
(a) Axial contrast-enhanced CT scan of the neck demonstrating bilateral parotid gland masses. Dominant ring-enhancing hypodense lesion of the left parotid gland measures 3.0 × 2.9 cm. (b) Coronally reconstructed CT scan with a prominent left cystic parotid mass and a smaller dense lesion of the right parotid gland. Few enlarged level 2 lymph nodes are noted bilaterally, ranging from 1.0 to 2.4 cm. Numerous non-pathologically enlarged jugular chain lymph nodes are visualized. (c) CT scan of the neck in sagittal reconstruction revealing a hypodense lesion with rim enhancement within the left superficial parotid gland.

**Figure 2.**
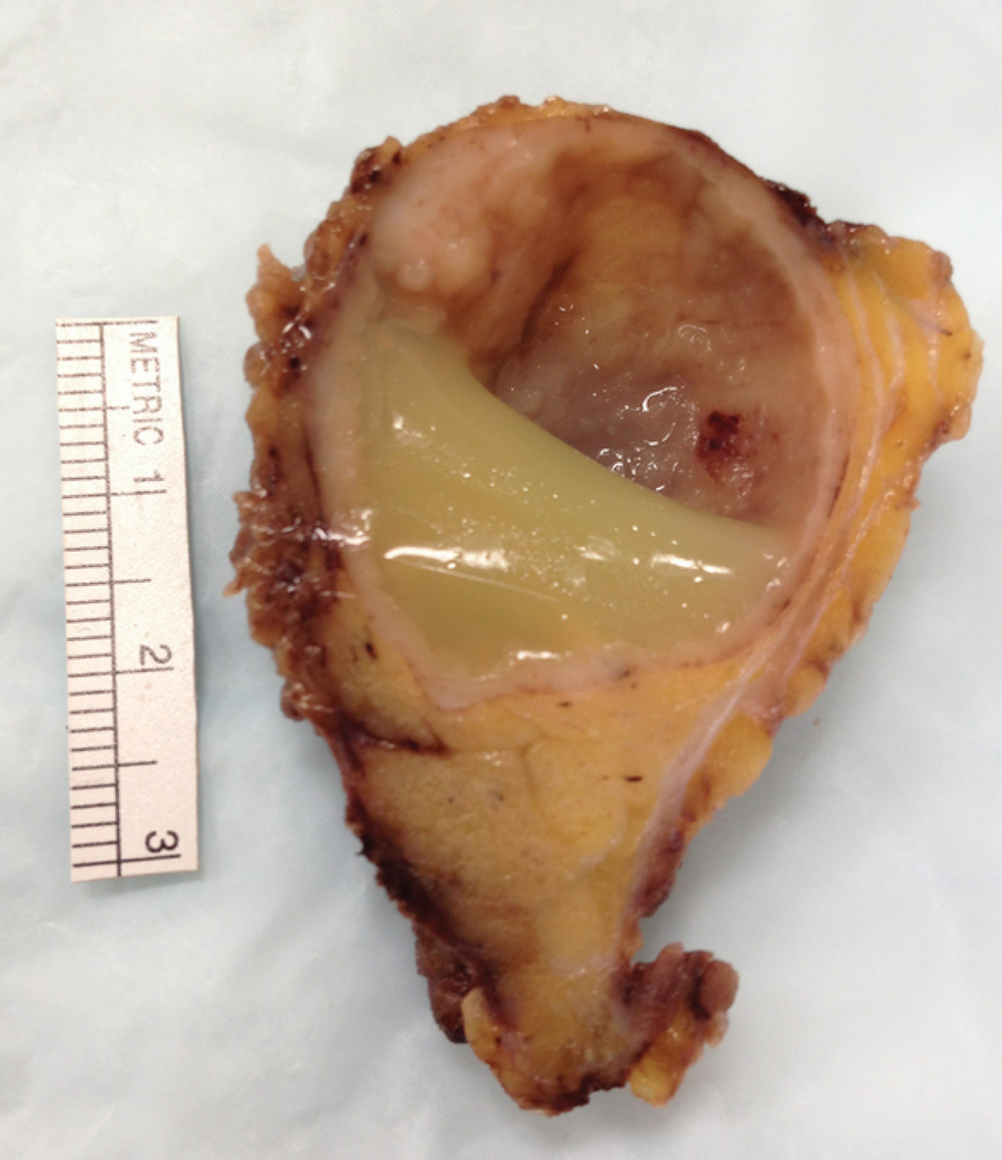
Gross specimen of left superficial parotidectomy with a 2.9 × 2.5 × 2.2cm cystic space containing clear yellow fluid. The wall of the cyst averages between 1.0 and 2.0 mm in thickness but is focally thickened up to 5.0 mm.

**Figure 3.**
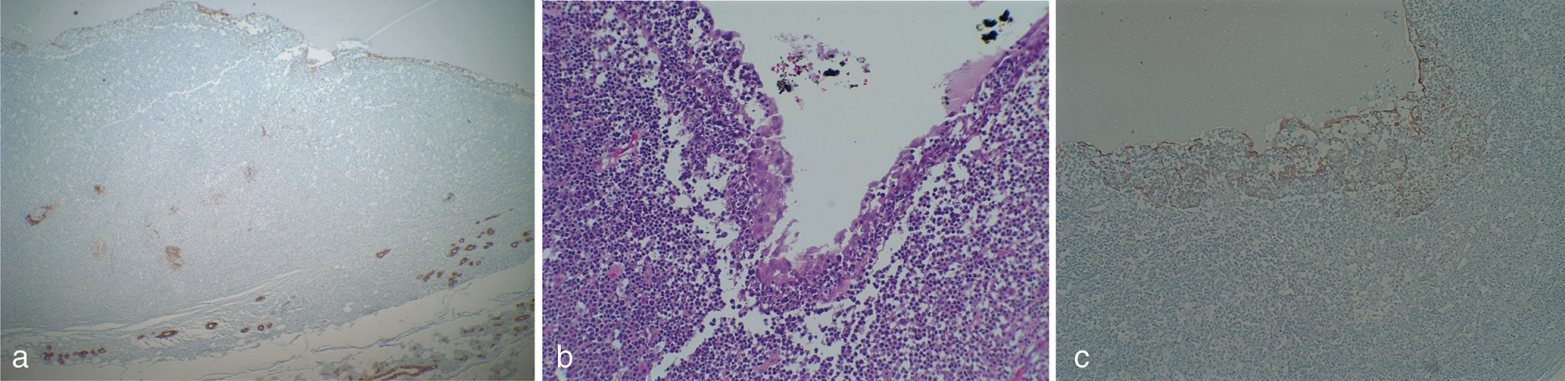
(a) Low power (40×) view of keratin stain showing lymphoepithelial complexes (brown islands) in the lymphoid tissue lining the cyst, confirming the diagnosis. (b) High power (400×) view of squamous lining of cyst on haematoxylin and eosin staining (large pink cells). (c) High power (400×) view showing keratin AE1/3 staining of squamous epithelial lining of lymphoepithelial cyst.

The patient is a 44-year-old immigrant male who presented to our institution with multiple masses in bilateral parotid glands, left greater than right. He had a history of neurocysticercosis, presumably owing to ingestion of uncooked pork in Mexico and had undergone a previous craniotomy with removal of the brain mass in Mexico. The patient had been vaccinated against tuberculosis, and subsequent work-up for systemic tuberculosis was negative. This new onset of bilateral parotid masses since his emigration to USA was presumed to represent cysticercosis lesions. A CT scan of the neck with i.v. contrast demonstrated bilateral parotid masses with a dominant, ring-enhancing, hypodense lesion in the left superficial parotid gland, which measured 3.0 × 2.9 cm (Figure 1). A left superficial parotidectomy was performed, and upon pathological analysis, the peripherally enhancing lesion grossly appeared as a large yellowish fluid-filled cyst (Figure 2). Micropathology revealed a squamous epithelium-lined cyst with lymphoepithelial complexes (brown islands) consistent with a lymphoepithelial cyst (Figure 3). This raised the concern of HIV infection, which was confirmed with serological studies.

## Discussion

BLEL, which were originally thought to be infrequently occurring lesions of the parotid gland characterized by lymphoid hyperplasia and concurrent cyst formation, have become more prevalent oral lesions in the setting of untreated HIV infection. Usually manifesting as painless enlargement of one or both parotid glands, BLEL have been described as a common index lesion for the diagnosis of HIV infection, and therefore must be entertained in the differential for cystic and mixed parotid pathology seen on imaging of any patient. The patient described had no diagnosis of HIV/AIDS upon presentation. His parotid swelling was initially thought to be a solitary, extracranial focus of known cysticercosis, but was ultimately found to be a pathologically proven lymphoepithelial cyst. Further serological analysis revealed HIV positivity, and the lymphoepithelial cyst served as the impetus for evaluation. Of note, a parotid focus of cysticercosis could also appear as a solitary, ring-enhancing lesion, but would exhibit predominance of neutrophils and macrophages on fine needle aspiration cytology as described by Veena et al^9^ in 2008. Hence, recognition of BLEL as potential harbingers of HIV infection through imaging is essential for diagnosis and subsequent timely and effective treatment.

## Learning points

1. Lymphoepithelial cysts should be a differential diagnostic consideration if cystic or solid bilateral intraparotid lesions are discovered in a patient with HIV, or where HIV is suspected.
2. Ancillary findings of cervical lymphadenopathy and proliferation of Waldeyer's ring should raise suspicion for BLEL in patients with bilateral parotid lesions.

## Consent

Consent is received from all patients at our teaching institution.
